# Development and Validation of SNP and InDel Markers for Pod-Shattering Tolerance in Soybean

**DOI:** 10.3390/ijms23042382

**Published:** 2022-02-21

**Authors:** Jeong-Hyun Seo, Sanjeev Kumar Dhungana, Beom-Kyu Kang, In-Youl Baek, Jung-Sook Sung, Jee-Yeon Ko, Chan-Sik Jung, Ki-Seung Kim, Tae-Hwan Jun

**Affiliations:** 1 Department of Southern Area Crop Science, National Institute of Crop Science, Rural Development Administration, Miryang 50424, Korea; next0501@korea.kr (J.-H.S.); sanjeev@korea.kr (S.K.D.); hellobk01@korea.kr (B.-K.K.); baekiy@korea.kr (I.-Y.B.); sjs31@korea.kr (J.-S.S.); kjeeyeon@korea.kr (J.-Y.K.); jung100@korea.kr (C.-S.J.); 2 Innovative Technology Department, FarmHannong, Ltd., Nonsan 33010, Korea; leehan26@snu.ac.kr; 3 Department of Plant Bioscience, Pusan National University, Miryang 50463, Korea; 4 Life and Industry Convergence Research Institute, Pusan National University, Miryang 50463, Korea

**Keywords:** Kompetitive Allele-Specific PCR, soybean, pod shattering tolerance, single nucleotide polymorphism, insertion/deletion, candidate gene, molecular marker

## Abstract

Pod-shattering causes a significant yield loss in many soybean cultivars. Shattering-tolerant cultivars provide the most effective approach to minimizing this loss. We developed molecular markers for pod-shattering and validated them in soybeans with diverse genetic backgrounds. The genes *Glyma.16g141200*, *Glyma.16g141500*, and *Glyma.16g076600*, identified in our previous study by quantitative trait locus (QTL) mapping and whole-genome resequencing, were selected for marker development. The whole-genome resequencing of three parental lines (one shattering-tolerant and two shattering-susceptible) identified single nucleotide polymorphism (SNP) and/or insertion/deletion (InDel) regions within or near the selected genes. Two SNPs and one InDel were converted to Kompetitive Allele-Specific PCR (KASP) and InDel markers, respectively. The accuracy of the markers was examined in the two recombinant inbred line populations used for the QTL mapping, as well as the 120 varieties and elite lines, through allelic discrimination and phenotyping by the oven-drying method. Both types of markers successfully discriminated the pod shattering-tolerant and shattering-susceptible genotypes. The prediction accuracy, which was as high as 90.9% for the RILs and was 100% for the varieties and elite lines, also supported the accuracy and usefulness of these markers. Thus, the markers can be used effectively for genetic and genomic studies and the marker-assisted selection for pod-shattering tolerance in soybean.

## 1. Introduction

Soybean is an economically important crop worldwide. The productivity of soybean is affected by various biotic and abiotic factors, including pod-shattering. The characterization of soybean productive units and the use of growth simulation models can help to identify some of the related factors of soybean productivity [1,2]. Pod-shattering is the opening of the mature pod along the dorsal or ventral suture with the dispersal of the seeds [3]. While it is essential for seed dispersal in wild species, in domesticated crops it can cause significant yield loss [4]. These losses vary from negligible to significant, depending on the harvest delay, the environmental conditions, and/or the tolerance of the variety [5,6,7].

Linkage mapping, commonly known as the quantitative trait locus (QTL) analysis [8], as well as genome-wide association studies (GWAS) [9] are mapping techniques that locate genomic regions associated with the quantitative variations for target traits. In soybean, the major genomic regions associated with the pod-shattering tolerance are on chromosome 16. These regions were identified through linkage mapping using diverse molecular markers, including restriction fragment length polymorphism markers [10], simple-sequence repeat (SSR) markers [11,12,13], and high-density single nucleotide polymorphism (SNP) markers [14]. A few minor QTLs have been mapped on chromosomes 2, 5, 10, 14, and 19 by linkage mapping [10,11]; on chromosomes 1, 4, 6, 8, 9, 11, 17, 18, and 20 through GWAS [15]; and on chromosomes 1, 5, 8, and 14 through linkage mapping using specific locus-amplified fragment sequencing [16]. We previously mapped major and minor QTLs on chromosome 16 and reported candidate genes for the pod-shattering tolerance in soybean [17].

Although pod-shattering is more than 90% heritable [18], pod-shattering in the field is greatly affected by diverse environmental factors, including humidity and temperature. To address the problems inherent in field evaluations, most pod-shattering assessments in soybeans are made using the oven-dry method [15,19,20]. However, the conventional method for evaluating pod-shattering (i.e., keeping the mature pods at room temperature for a few days to equalize the moisture content among samples) is time-consuming and labor-intensive. Moreover, this technique is applicable only for mature pods (R8 stage) at later growth stages. Therefore, an efficient and accurate selection technique, such as molecular markers that can be applied at all growth stages, is needed to determine the pod-shattering tolerance versus susceptibility in soybean breeding programs.

Among various molecular markers, Kompetitive Allele-Specific PCR (KASP) and insertion/deletion (InDel) markers have been used widely for many crops because they are simple, reproducible, stable, accurate, fast, and are low-cost [21,22,23,24]. KASP assays for the marker-assisted selection (MAS) of the egusi trait in the watermelon [24], the flowering gene in lentils [22], and the white tip nematode (*Aphelenchoides besseyi*) in rice [25] have been developed and applied to diverse genetic backgrounds. In soybean, KASP assays have been employed for a few traits, including soybean cyst nematode resistance [26,27], frogeye leaf spot resistance [28], and *Fusarium graminearum* resistance [29]. However, there are few reports of selection markers for the pod-shattering tolerance in soybean [30,31]. We designed SNP and/or InDel markers and validated them in populations with diverse genetic backgrounds. These markers could be used by MAS for initial germplasm selection and for developing soybean cultivars with a pod-shattering tolerance.

## 2. Results

### 2.1. Development of KASP Markers

For KASP markers, we selected the SNPs or InDel regions in the candidate genes of the major QTL [17] by comparing the DNA sequences of the tolerant parent Daewonkong (DW) and the susceptible parents Tawonkong (TW) and Saeolkong (SO). Of the five candidate genes from within the major QTL, three genes had five SNPs. For the two genes *Glyma.16g141200* and *Glyma.16g141500* (Supplementary Figures S1 and S2) on chromosome 16, these SNPs had no other polymorphic variations within 100 bp upstream or downstream.

The first marker, *KASP-PS-1* for *Glyma.16g141200*, had a single bp deletion (TC > T) at 29,916,524 bp on chromosome 16. The tolerant parent DW and the reference genome (reference, version) had a “C” allele in the target region, whereas the susceptible parents TW and SO had the deletion (Table 1, Supplementary Figure S1). The second marker, *KASP-PS-2* for *Glyma.16g141500*, had a SNP variant (T > G) at 29,966,815 bp on chromosome 16. The tolerant parent DW and the reference genome had the “T” allele in the target region, whereas the susceptible parents had the “G” allele. We developed the KASP marker with allele-specific primers and common primers in each SNP (Table 1, Supplementary Figure S2).

### 2.2. Development of InDel Markers

Previously, we reported a novel candidate gene *Glyma.16g076600* related to abscisic acid (ABA) catabolism [17]. There were four SNPs, one insertion, and one deletion in the target region, causing amino acid changes (Table 2). This gene had a translational stop codon (nonsense) mutation, which would stop protein expression from the gene. We designated the InDel marker for the 18-bp insertion region of the candidate gene *Glyma.16g076600* (Supplementary Figure S3). DW had the additional sequence differences (the DW-specific allele) compared to the reference genome TW and SO (Table 2).

### 2.3. Validation of KASP and InDel Markers in RIL Populations

**Figure 1:**
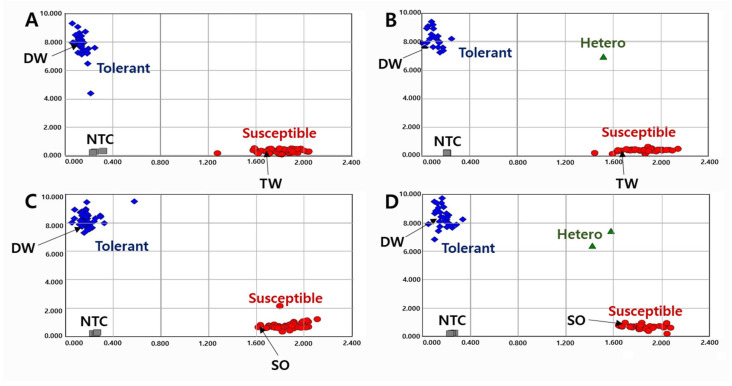
Allelic discrimination of RIL populations using the *KASP-PS-1* marker. The upper two ((**A**): 91 RILs and 2 parents, (**B**): 63 RILs and 2 parents because up to 96 genotypes were accommodated in a single experimental set-up) figures for 154 RILs of DT combination (DW × TW population) and the lower two ((**C**): 91 RILs and 2 parents, (**D**): 62 RILs and 2 parents) figures for 153 RILs of DS combination (DW × SO population). NTC: No template control, DW: Daewonkong, TW: Tawonkong, and SO: Saeolkong.

**Figure 2:**
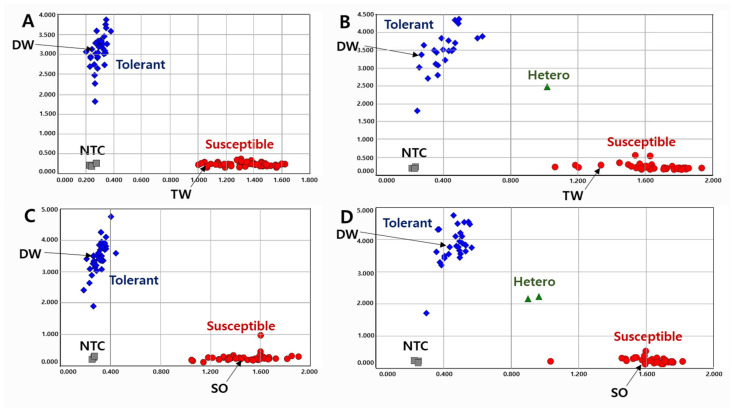
Allelic discrimination of RIL populations using the *KASP-PS-2* marker. The upper two figures ((**A**): 91 RILs and 2 parents, (**B**): 63 RILs and 2 parents because up to 96 genotypes were accommodated in a single experimental set-up) for 154 RILs of DT combination (DW × TW population) and the lower two figures ((**C**): 91 RILs and 2 parents, (**D**): 62 RILs and 2 parents) for 153 RILs of DS combination (DW × SO population). NTC: No template control, DW: Daewonkong, TW: Tawonkong, and SO: Saeolkong.

To validate the KASP markers in RILs, we genotyped the lines with two KASP markers and determined the pod-shattering ratio in 154 RILs from the DW × TW (DT population) and 153 RILs from the DW × SO (DS population). In the scatter plot, the RILs, shown by the blue spot, had a tolerance allele from DW, while the RILs indicated by the red spot had a susceptibility allele (Figure 1 and Figure 2). The results showed that the pod-shattering-tolerant parent DW and the susceptible parents TW and SO were successfully distinguished by both markers. Similarly, the tolerant lines and susceptible lines were clearly distinguished according to their genotypes. Among the RILs, 56 lines had the tolerance allele and 88 lines had the susceptibility allele, and the others were undetermined or had a heterozygous allele in the DT population. Similarly, 72 lines had the tolerance allele, 78 lines had the susceptibility allele, and the others had a heterozygous allele in the DS population (Figure 1 and Figure 2).

In the DT population, the lines with the tolerance genotype had 1.6%, 5.1%, and 9.0% pod-shattering ratios for 24, 48, and 72 h of oven-drying, respectively. Those with the susceptible genotype had high ratios of 25.9%, 55.0%, and 75.1% for the same periods of drying, respectively. Similarly, in the DS population, 4.9%, 12.3%, and 18.3% of the pods of tolerant lines were shattered after 24, 48, and 72 h, respectively. Shattered pods of susceptible lines were 44.0%, 80.4%, and 86.3% for the same periods of drying, respectively (Table 3).

As expected for the InDel marker in the RILs, the tolerant parent and susceptible parents gave different product sizes (DW, 144 bp; TW and SO, 126 bp) for the InDel marker (Supplementary Figure S4). Among the RILs, 69 lines had the tolerance allele, 74 lines had the susceptibility allele, and the others had undetermined or heterozygous alleles in the DT population. Similarly, 74 lines had the tolerance allele, 76 lines had the susceptibility allele, and the others had undetermined or heterozygous alleles in the DS population (Supplementary Figure S4).

In the DT population, 7.4%, 20.3%, and 27.9% of the pod-shattering ratios were found for the genotypes with the DW allele after 24, 48, and 72 h of drying, respectively. However, the lines with the TW or SO allele produced 24.7%, 49.5%, and 69.0% of the pod-shattering ratios for the same periods of drying, respectively (Table 3). Likewise, in the DS population, 7.6%, 19.7%, and 26.4% of the pod-shattering ratios were observed in the tolerant lines, whereas the susceptible lines gave 43.0%, 75.9%, and 81.2% of the pod-shattering ratios after 24, 48, and 72 h of oven-drying, respectively (Table 3).

### 2.4. Validation of KASP and InDel Markers in Diverse Varieties and Elite Lines

**Figure 3:**
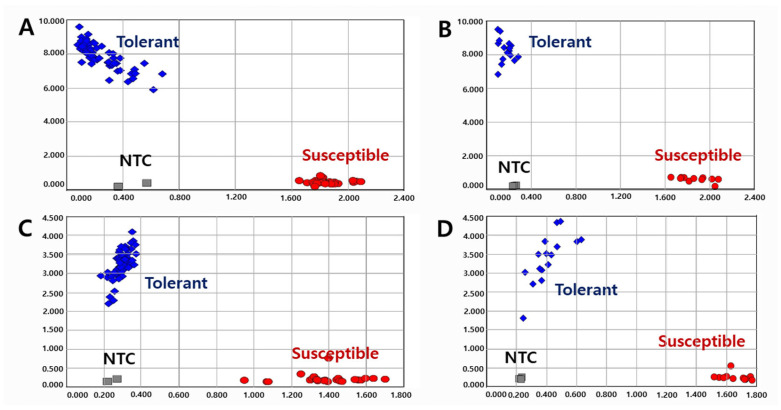
Allelic discrimination of 120 varieties and elite lines using *KASP-PS-1* ((**A**): 93 and (**B**): 27) and *KASP-PS-2* ((**C**): 93 and (**D**): 27) markers. NTC: No template control.

A total of 120 varieties and elite lines with diverse genetic backgrounds were tested to validate the KASP (Figure 3) and InDel (Supplementary Figure S5) markers. Although the efficiency of the InDel marker was lower than that of KASP, both markers successfully distinguished the pod-shattering-tolerant and -susceptible genotypes. In the KASP marker analysis, 1.4%, 3.6%, and 5.7% of the pod-shattering ratios were seen in the tolerant genotypes after 24, 48, and 72 h of oven-drying, respectively, while 44.6%, 76.2%, and 85.1% of the pod-shattering ratios were observed in the susceptible genotypes, respectively (Table 4). In the case of the InDel marker, the varieties and elite lines with the tolerant genotype showed 4.7%, 11.6%, and 14.4% of the pod-shattering ratios after 24, 48, and 72 h of oven-drying, respectively. However, the lines representing the susceptible genotype had 15.0%, 26.1%, and 30.7% pod-shattering ratios after 24, 48, and 72 h of oven-drying, respectively (Table 4).

### 2.5. Association between Allelic Combination and Phenotypes

**Figure 4:**
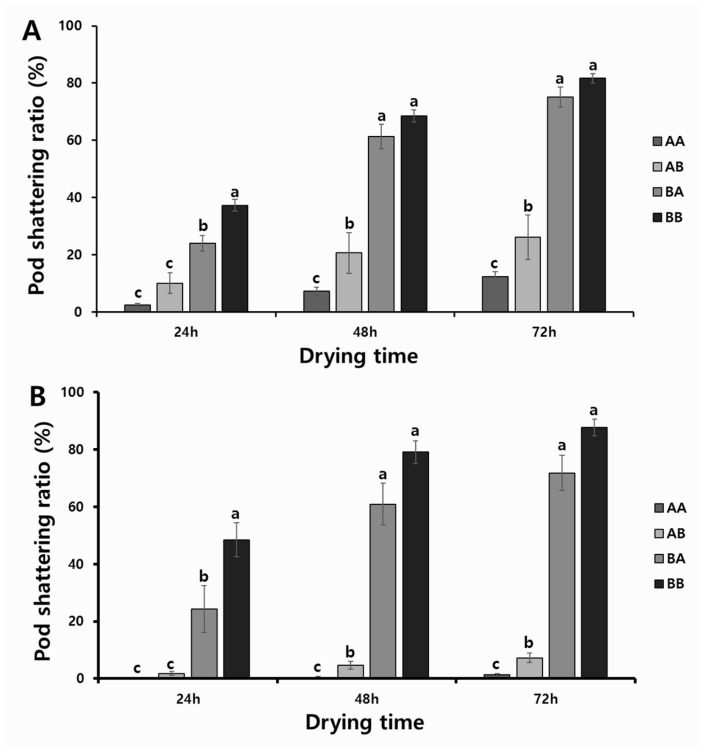
Phenotypic variation for pod-shattering ratio according to their genotypes: (**A**) two RIL populations and (**B**) varieties and elite lines. AA: tolerant genotypes in both markers; AB: tolerant genotypes in KASP marker but susceptible genotype in InDel marker; BA: susceptible genotypes in KASP marker but tolerant genotypes in InDel marker; and BB: susceptible in both markers. Standard errors are used to depict error bars. The letters (a–c) above bar diagrams represent significant differences at *p* < 0.05 by Duncan’s multiple range test.

To determine the associations between the allelic combinations obtained with KASP and InDel markers (A, tolerant; B, susceptible) and phenotypic variations, we divided 307 RILs from the two populations into four groups; i.e., the tolerant genotypes in both markers (AA); the tolerant genotypes with the KASP marker, but also the susceptible genotypes with the InDel marker (AB); the susceptible genotypes with the KASP marker, but also the tolerant genotypes with the InDel marker (BA); and the susceptible genotypes with both markers (BB). As a result, we discovered significant differences in the pod-shattering ratio depending on the allelic combinations of RILs. The RILs with the allele type AA had 2.4%, 7.2%, and 12.2% of the pod-shattering ratios after 24, 48, and 72 h of oven-drying, respectively, while allele type AB had 10.0%, 20.6%, and 26.1% of the pod-shattering ratios after 24, 48, and 72 h of oven-drying, respectively. Allele type BA had 24.0%, 61.3%, and 75.1% of the pod-shattering ratios after 24, 48, and 72 h of oven-drying, respectively, and allele type BB had 37.2%, 68.5%, and 81.7% of the pod-shattering ratios after 24, 48, and 72 h of oven-drying, respectively (Figure 4A). The varieties and elite lines also showed similar patterns of pod-shattering after 24, 48, and 72 h of oven-drying to that of RILs (AA, 0.2%, 0.5%, and 1.3%; AB, 1.8%, 4.6%, and 7.2%; BA, 24.3%, 60.9%, and 71.8%; and BB, 48.5%, 79.1%, and 87.7%) (Figure 4B).

The prediction accuracy of the KASP marker was very high, with 81.7% to 88.2% in the RIL populations and 91.3% to 95.5% in the varieties and elite lines. Considering both types of markers in the strains with tolerant genotypes, for the RIL populations, the prediction accuracy was 83.4% to 90.9%, and for the varieties and elite lines, the prediction accuracy was 100%, which was higher than the KASP marker alone (Table 5).

### 2.6. Haplotype Analysis of Candidate Genes

**Figure 5:**
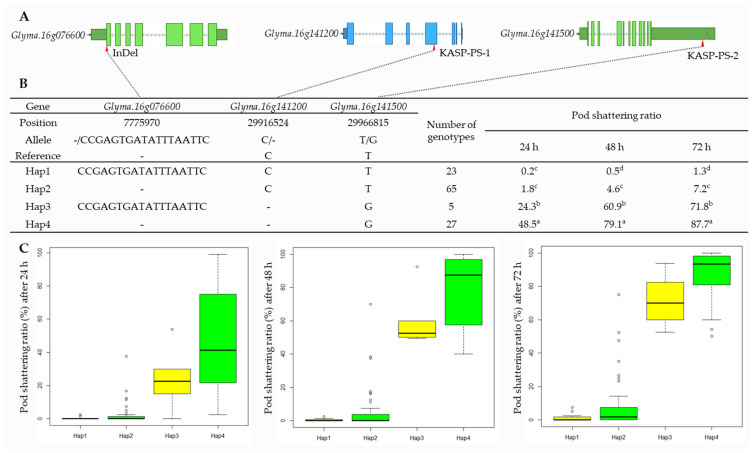
Haplotype analysis of *Glyma.16g076600*, *Glyma.16g141200*, and *Glyma.16g141500*. (**A**) Schematic representation of gene structure and InDel and SNPs positions. (**B**) Results of haplotype analysis. Hap: Haplotype. Superscript letters (a-d) represent significant differences at *p* < 0.05. (**C**) Haplotype variation analysis. Colored boxes indicate the pod-shattering ratio of soybean genotypes in each Hap.

The results of the haplotype analysis for *Glyma.16g076600*, *Glyma.16g141200*, and *Glyma.16g141500* are shown in Figure 5. For *Glyma.16g076600*, there were four SNPs, one insertion (18 bp) at 7,775,970 bp that was used to develop the InDel marker, and one deletion in the target region (Table 2, Figure 5, and Supplementary Figure S3). *Glyma.16g141200* had a single bp deletion (TC > T) at 29,916,524 bp. *Glyma.16g141500* had an SNP variant (T > G) at 29,966,815 bp that was considered while developing KASP markers (Table 1, Figure 5, and Supplementary Figures S2 and S3). The haplotype analysis of the 120 varieties and elite lines showed that the InDel and SNPs were divided into four haplotypes (Haps), with the maximum pod-shattering variations of 48.3, 78.6, and 86.4 at 24, 48, and 72 h of oven-drying, respectively, between Haps 1 and 4 (Figure 5B).

## 3. Discussion

Pod-shattering causes a significant yield loss in soybeans. The selection of elite breeding lines with good agronomic traits, including pod-shattering tolerance, is essential for soybean breeding programs around the world. Molecular markers offer an effective and efficient approach for studying pod-shattering, as well as for breeding programs.

Several studies have identified molecular markers targeting genes on chromosome 16, the major genomic region associated with the pod-shattering phenotype [10,17,32]. Three InDel markers (SRM0, SRM1, and SRM2) were developed for the *qPDH1* locus [13]. The one SNP variation in the coding region of *Glyma16g25580* (Wm82.a1.v2) was developed as a Pdh1 Simple Probe real-time PCR assay for predicting pod-shattering [4]. The SNPs within *Glyma.16g141600* on chromosome 16 also have significant associations with pod-shattering [32]. However, for a highly accurate selection of soybean genotypes, we need additional molecular markers for pod-shattering tolerance [31]. Therefore, we wanted to confirm the candidate genes and develop additional allele-specific markers related to the pod-shattering tolerance from an elite cultivar DW with strong tolerance to pod-shattering.

It has been reported that the inheritances of pod shattering in soybean are controlled by additive gene action [33,34], which is the effect of multiple genes with a linear or additive fashion [35]. Previous studies have suggested that the genetic variation in pod-shattering in soybean is controlled by partial dominance [36], dominant epistasis [37], dominance, or partial dominance [38]. Bhor et al. [39] found that two major genes with inhibitory epistasis were responsible for the mode of inheritance of pod-shattering. A single major gene may not be enough to differentiate soybean genotypes for pod-shattering. Han et al. [40] reported no difference between soybean cultivars Heihe 43 (tolerant pod-shattering) and Heihe 18 (susceptible to pod-shattering) using the major pod-shattering tolerance gene *SHATTERING1-5* [41]. However, the cultivars were successfully differentiated by *Glyma.05g225900* and *Glyma.05g227400*. Therefore, the identifications of additional genes and the use of molecular markers linked with the genes would be highly useful for genetic studies and breeding programs in soybean. In the present study, we developed KASP markers for *Glyma.16g141200* and *Glyma.16g141500*, as well as an InDel marker for *Glyma.16g076600*, which are all candidate genes for pod-shattering that we identified within the QTLs detected previously [17]. *Glyma.16g076600* is a novel candidate gene found in our previous study [17]. In addition, we evaluated the efficiency of the markers using soybean genotypes with diverse genetic backgrounds that are tolerant or susceptible to pod-shattering.

The functions of the three candidate genes involved in the pod-shattering mechanisms have been studied previously. It has been suggested that pod-shattering involves a bZIP transcription factor related to *Glyma.16g141500* (bZIP transcription factor bZIP117), located in a major pod-shattering QTL (*qPDH1*) in soybean [14]. The bZIP proteins play a role in the expression of the proline dehydrogenase (ProDH) gene in *Arabidopsis* [42]. Kavi Kishor et al. [43] reported that proline is an important source of the cell wall matrix. Proline-based signaling proteins are also important for the cell wall of *Arabidopsis thaliana* [44]. The increased expression of ProDH2 in senescent leaves and in the abscission zone of floral organs implicates a bZIP transcription factor in pod-shattering [45].

The pod-shattering tolerance in soybean may result from excessive secondary cell wall deposition and lignification in the outer part of the suture [41]. This is supported by a study in common vetch, in which high-throughput RNA sequencing was used to evaluate global transcriptome variations associated with the pod ventral sutures of accessions for the shattering-susceptible and shattering-resistant strains [46]. A similar study demonstrated that the shattering-resistant varieties of common vetch did not have an abscission zone in the bundle sheath; thus, this favors the development of strong sutures [47]. The expression of a gene associated with stem bolting in *Arabidopsis* indicates that members of the bZIP family may be involved in secondary cell wall thickening [48]. Moreover, several TGA-type bZIP genes may control the expression of the genes involved in abscission [49]. The upregulation of 11 bZIP genes in the abscission zones of *Elymus nutans* supports their role as positive regulators in abscission [50]. Similar to the involvement of *Glyma.16g141500* in abscission, protein phosphatase 2C, which is related to *Glyma.16g141200*, may also function in abscisic acid signal transduction in *Arabidopsis thaliana* [51]. *Glyma.16g076600*, a homolog of *AT4G19230* in *Arabidopsis*, is a member of the CYP707A gene family that encodes a protein related to ABA catabolism [52]. Plant hormones, such as ABA, may regulate silique dehiscence in *Arabidopsis* and *Brassica* [53].

We evaluated the KASP and InDel markers for the pod-shattering phenotypes using the oven-drying method in 307 RILs and 120 varieties and elite lines. The prediction accuracy of the KASP marker was very high, with 81.7% to 88.2% in the RIL populations, and 91.3% to 95.5% in the varieties and elite lines. The lower prediction accuracy in the RIL populations may be due to a recombination in the RILs (although they were F_7:8_), and these results were similar to the results of Kim et al. [31]. The prediction accuracy of this study is comparable to a previous study [31]. The slightly higher values obtained in the study [31] might be due to their use of fewer RILs of the shattering-tolerant breeding lines. Using both types of markers could increase the prediction accuracy in soybean lines, compared to using KASP markers alone. Adding markers from the additional candidate genes could increase the prediction accuracy. The haplotype analysis of candidate genes revealed distinct groups of soybean genotypes for the pod-shattering tolerance. The three genes used here as markers for pod-shattering in soybean, as well as the KASP and InDel markers developed in this study, could be used in MAS to improve the pod-shattering tolerance in soybean breeding programs.

## 4. Materials and Methods

A summary of the study design has been provided as a schematic diagram (Supplementary Figure S6).

### 4.1. Plant Materials

The QTLs and candidate genes for pod-shattering tolerance were identified using two RIL populations (F_7:8_) derived from the cross between a pod-shattering-tolerant cultivar Daewonkong (DW) and the pod-shattering-susceptible cultivars, Tawonkong (TW) and Saeolkong (SO), as described previously [17,54] (Supplementary Figure S7). Populations consisting of 154 RILs (DW × TW; DT), 153 RILs (DW × SO; DS), and 120 varieties and elite lines were used to validate the KASP and InDel markers for the candidate genes *Glyma.16g141200*, *Glyma.16g141500*, and *Glyma.16g076600*. Two RIL populations, as well as 120 soybean varieties and elite lines used in this study, were developed in our institute according to the cultivation methods of the Agricultural Science Technology Standards for Investigation (Rural Development Administration, Jeonju, Korea) and they comply with the guidelines and legislation of Korea. The varieties and elite lines were selected based on their economic values and cultivation practices in Korea. The panel of the 120 varieties and elite lines includes 86 pod-shattering-tolerant and 34 pod-shattering-susceptible soybeans. All the plant materials are deposited at the National Institute of Crop Science, Rural Development Administration, Miryang, Korea, and can be obtained on reasonable request after meeting the institutional obligations.

### 4.2. Evaluation of Pod-Shattering Tolerance

The pod-shattering tolerance for 400 genotypes (307 RILs, 120 varieties, and elite lines) was calculated, as described previously [17,54]. In brief, mature pods were harvested at the R8 stage, stored at room temperature for a week, oven-dried (40 °C) for 24, 48, or 72 h, and the number of shattered pods were counted. The pod-shattering ratio = (number of shattered pods/total number of pods) × 100 (%).

### 4.3. DNA Extraction from the Parental Lines and Whole-Genome Resequencing

Genomic DNA was extracted from the young trifoliate leaves of three parental cultivars (Daewonkong, Tawonkong, and Saeolkong) of the RIL populations using a DNA extraction kit (Exgene Plant SV Miniprep Kit; GeneAll, Seoul, Korea) following the manufacturer’s instructions. The concentration and quality of the extracted DNA were determined with a NanoDrop 2000 spectrophotometer (Thermo Fisher Scientific, Waltham, MA, USA). The Illumina Hi-seq 4000 whole-genome resequencing of the parental cultivars (Macrogen Inc., Seoul, Korea) revealed the SNP and/or InDel regions, as compared to the reference genome of *Glycine max* (Wm82.a2.v1) [55]. The sequences were visualized using the Golden Helix Genome Browser v 3.0.0 software (Golden Helix, Bozeman, MT, USA).

### 4.4. Molecular Marker Development and Genotyping

The genomic DNA from 307 RILs of two populations, as well as 120 varieties and elite lines, was extracted, as described above. The KASP markers for the target SNPs were developed with allele-specific primers and common primers. The KASP markers were genotyped by using the ABI7300 system (ABI, Foster City, CA, USA) and were analyzed with 7300 system SDS RQ study software (Thermo Fisher Scientific, Waltham, MA, USA). The PCR (polymerase chain reaction) conditions for genotyping with the KASP marker were as follows: 94 °C for 15 min; 10 cycles of 94 °C for 20 s, 61 °C for 1 min; and 26 cycles of 94 °C for 20 s; followed by 55 °C for 1 min. The InDel marker for the target sequence was generated using the Primer3 program (http://bioinfo.ut.ee/primer3-0.4.0, Accessed 19 October 2021). The InDel marker was genotyped by capillary electrophoresis (QIAxcel, Qiagen, Hilden, Germany). The PCR conditions for genotyping with the InDel marker were as follows: 95 °C for 5 min; 35 cycles of 95 °C for 20 s, 55 °C for 1 min, 72 °C for 1 min; followed by 72 °C for 5 min.

### 4.5. Determination of Prediction Accuracy

The prediction accuracy was evaluated following the method by Kim et al. [31]. The susceptibility accuracy was calculated as the proportion of the lines with susceptible phenotypes, compared to the lines with susceptibility alleles. Similarly, the tolerance accuracy was calculated as the proportion of the lines with resistant phenotypes compared to the lines with resistance alleles. The prediction accuracy was calculated as the mean of susceptible accuracy and tolerant accuracy.

### 4.6. Haplotype Analysis

The haplotype analysis for *Glyma.16g076600*, *Glyma.16g141200*, and *Glyma.16g141500* was carried out to assess genetic variations among soybean genotypes using the methods by Zhang et al. [56] with some modifications. In 120 varieties and elite lines panels, one InDel and two SNPs markers were used for the haplotype analysis. The average score and genotype count were determined from the pod-shattering ratio, and the haplotypes that were significantly associated with pod shattering were identified.

### 4.7. Statistical Analysis

R software V 4.1.1 (R Foundation for Statistical Computing, Vienna, Austria) was used to determine the analysis of variance and compare the phenotypic variations among the genotypes of RIL populations, varieties, and elite lines.

## 5. Conclusions

Two KASP markers and one InDel marker were developed based on our previous studies on QTL mapping and the analysis of candidate genes for pod-shattering tolerance using two RIL populations. The whole-genome resequencing of three parental lines revealed SNP and/or InDel regions based on comparisons to the Williams_82 reference genome (ver. 2.1). The markers were validated in 307 RILs, 120 varieties, and elite lines through allelic discrimination. The validity of the markers in these lines was further supported by the pod-shattering ratios and the high prediction accuracy of these results. The consistency of the results in diverse genetic backgrounds indicates that the markers can be used to select pod-shattering tolerant genotypes. These markers may also provide insights into the molecular mechanisms underlying the pod-shattering tolerance in soybean.

## Figures and Tables

**Table 1:** Summary of SNP information on the candidate genes of the major quantitative trait locus [17].

Gene Model	SNP Position (bp)	Polymorphic Site	SNP Information	Amino Acid Change
*Glyma.16g141200*	29,916,524	3′ UTR	Deletion (TC > T)	-
*Glyma.16g141500*	29,964,216	5′ UTR	A > C	-
*Glyma.16g141500*	29,966,815	5′ UTR	T > G	-
*Glyma.16g141600*	29,970,894	Coding region	T > C Nonsynonymous	(Asp > Gly)
*Glyma.16g141600*	29,970,957	Coding region	T > C Nonsynonymous	(Asn > Ser)

**Table 2:** Summary of SNP/InDel information on the candidate gene of the minor quantitative trait locus [17].

Gene Model	Position (bp)	Polymorphism Site	Codon Change	Amino Acid Change
*Glyma.16g076600*	7,775,892	Coding region	C > T Nonsynonymous	(Glu > Lys)
	7,775,945	Coding region	T > A Nonsynonymous	(Lys > Met)
	7,775,948	Coding region	A > G Nonsynonymous	(Ile > Thr)
	7,775,970	Coding region	Insertion (18 bp)	Stop gained and disruptive inframe insertion
	7,776,045	Coding region	C > T Nonsynonymous	(Met > Ile)
	7,777,575	Coding region	Deletion (3 bp) Nonsynonymous	Inframe deletion (Asn > -)

**Table 3:** Phenotypic (pod-shattering ratio) variations in the recombinant inbred line populations according to the genotypes.

Marker Type	Population	Genotype	Pod Shattering Ratio (%)		
			24 h	48 h	72 h
KASP	DT	P1	1.6a	5.1a	9.0a
		P2	25.9b	55.0b	75.1b
	DS	P1	4.9a	12.3a	18.3a
		P2	44.0b	80.4b	86.3b
InDel	DT	P1	7.4a	20.3a	27.9a
		P2	24.7b	49.5b	69.0b
	DS	P1	7.6a	19.7a	26.4a
		P2	43.0b	75.9b	81.2b

DT: Daewonkong × Tawonkong, DS: Daewonkong × Saeolkong. ‘P1’ indicates that the target allele is the same as the tolerant parent ‘Daewonkong’, ‘P2’ indicates that the target allele is the same as susceptible parents ‘Tawonkong’ and ‘Saeolkong’. Pod-shattering ratio is the mean value at the different drying periods (24, 48, and 72 h). Different letters within the same column indicate significant differences (*p* < 0.05).

**Table 4:** Phenotypic (pod-shattering ratio) variations in the 120 varieties and elite lines according to the genotypes.

Marker Type	Genotype	Pod-Shattering Ratio (%)		
		24 h	48 h	72 h
KASP	P1	1.4a	3.6a	5.7a
	P2	44.6b	76.2b	85.1b
InDel	P1	4.7a	11.6a	14.4a
	P2	15.0b	26.1b	30.7b

‘P1’ indicates that the target allele is the same as that of the tolerant parent ‘Daewonkong’, ‘P2’ indicates that the target allele is the same as that of the susceptible parents ‘Tawonkong’ and ‘Saeolkong’. Pod-shattering ratio is the mean value at the different drying periods (24, 48, and 72 h). Different letters followed by the values within the same column indicate significant differences (*p* < 0.05).

**Table 5:** The prediction accuracy between the genotypes and phenotypes of recombinant inbred lines (RILs), varieties, and elite lines for three drying periods.

Genotypes	RIL Populations						Varieties and Elite Lines					
	24 h		48 h		72 h		24 h		48 h		72 h	
	Tol	Sus	Tol	Sus	Tol	Sus	Tol	Sus	Tol	Sus	Tol	Sus
X	116	12	101	27	82	46	84	4	80	8	73	12
Y	31	135	4	162	1	165	4	27	0	31	0	31
Accuracy (%)	86.0		88.2		81.7		91.3		95.5		92.9	
AA	101	7	90	18	73	35	22	0	22	0	22	0
BB	22	109	2	129	1	130	0	26	0	26	0	26
Accuracy (%)	88.4		90.9		83.4		100		100		100	

Genotypes, X: tolerant genotype for KASP marker; Y: susceptible genotype for KASP marker; AA: tolerant genotype in both types of markers; and BB: susceptible genotype in both types of markers. Tol: number of tolerant lines (shattered pods ≤ 10%) and Sus: number of susceptible lines (shattered pods > 10%). Accuracy: prediction accuracy is the possibility that tolerant genotypes showed a tolerant phenotype and susceptible genotypes showed a susceptible phenotype.

## Data Availability

The data sets generated in this study are included in this published article and its Supplementary Materials.

## Supplementary Materials

The following supporting information can be downloaded at: https://www.mdpi.com/article/10.3390/ijms23042382/s1.
