# Supplementary material for: Development and Validation of SNP and InDel Markers for Pod-Shattering Tolerance in Soybean
Source: Int J Mol Sci. 2022 Feb 21;23(4):2382. doi: 10.3390/ijms23042382 (PMC8880809; doi:10.3390/ijms23042382)
Supplement: Supplementary file 1 [file ijms-23-02382-s001.zip › ijms-1571268-SI.pdf]

International Journal of
Molecular Sciences

MDPI

Article

Development and Validation of SNP and InDel Markers for Pod-Shattering Tolerance in Soybean

Jeong-Hyun Seo [1,†], Sanjeev-Kumar Dhungana [1,†], Beom-Kyu Kang [1], In-Youl Baek [1], Jung-Sook Sung [1], Jee-Yeon Ko [1], Chan-Sik Jung [1], Ki-Seung Kim [2] and Tae-Hwan Jun [3,4,*]

[1] Department of Southern Area Crop Science, National Institute of Crop Science, Rural Development Administration, Miryang 50424, Korea; next0501@korea.kr (J.-H.S.); sanjeev@korea.kr (S.-K.D.); hellobk01@korea.kr (B.-K.K.); baekiy@korea.kr (I.-Y.B.); sjs31@korea.kr (J.-S.S.); kjeeyeon@korea.kr (J.-Y.K.); jung100@korea.kr (C.-S.J.)
[2] Innovative Technology Department, FarmHannong, Limited, Nonsan 33010, Korea; leehan26@snu.ac.kr
[3] Department of Plant Bioscience, Pusan National University, Miryang 50463, Korea
[4] Life and Industry Convergence Research Institute, Pusan National University, Miryang 50463, Korea
* Correspondence: thjun76@pusan.ac.kr
† These authors contributed equally to this work.

Citation: Seo, J.-H.; Dhungana, S.-K.; Kang, B.-K.; Baek, I.-Y.; Sung, J.-S.; Ko, J.-Y.; Jung, C.-S.; Kim, K.-S.; Jun, T.-H.Development and Validation of SNP and InDel Markers for Pod-Shattering Tolerance in Soybean. *Int. J. Mol. Sci.* **2022**, *23*, 2382. https://doi.org/10.3390/ijms23042382

Academic Editors: Andrés J. Cortés; Hai Du

Received: 10 January 2022
Accepted: 18 February 2022
Published: 21 February 2022

Publisher's Note: MDPI stays neutral with regard to jurisdictional claims in published maps and institutional affiliations.

Copyright: © 2022 by the authors. Submitted for possible open access publication under the terms and conditions of the Creative Commons Attribution (CC BY) license (https://creativecommons.org/licenses/by/4.0/).

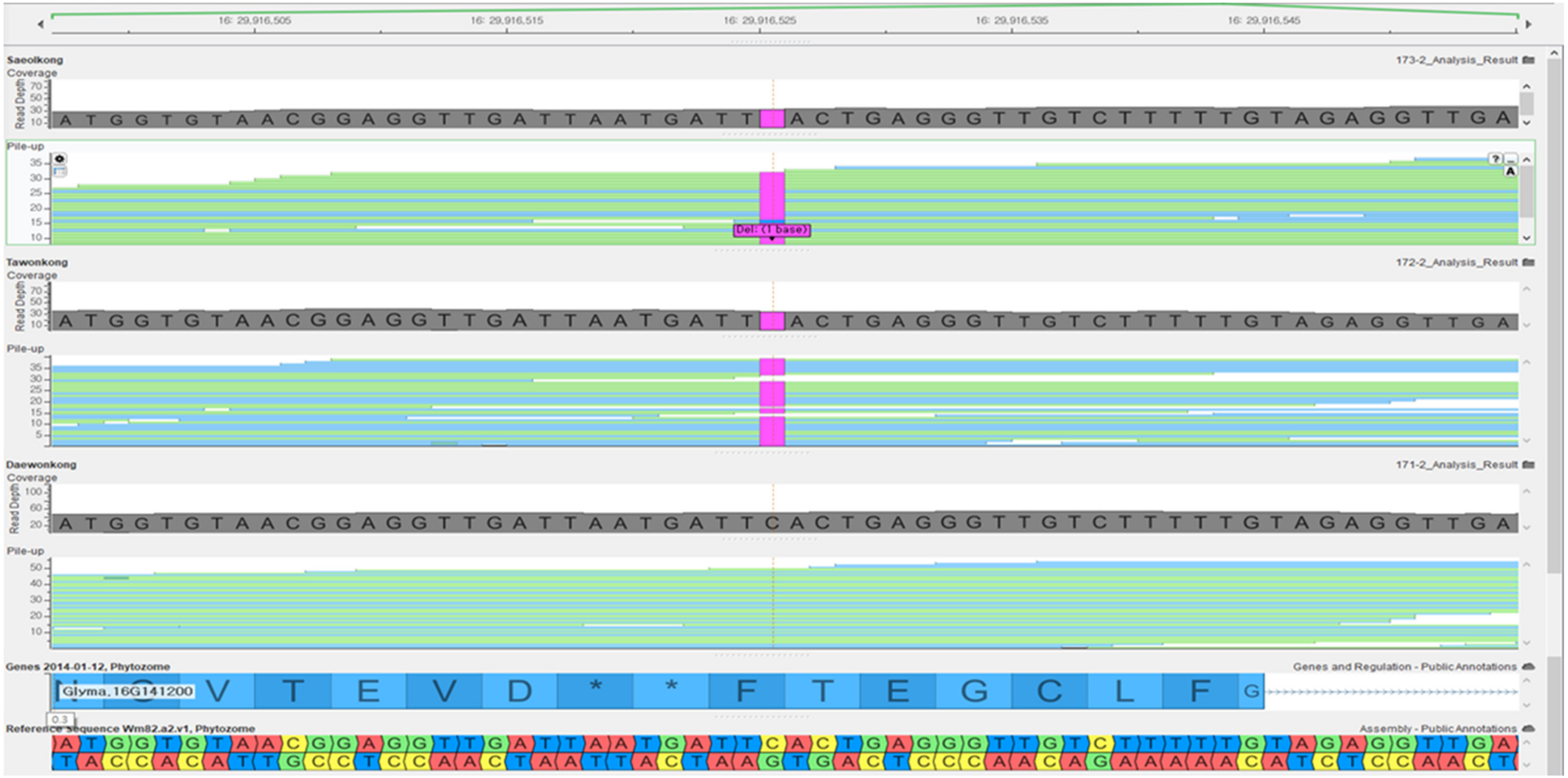

Figure S1. Target sequence of the *KASP-PS-1* marker (*Glyma.16g141200*, 1 bp deletion).

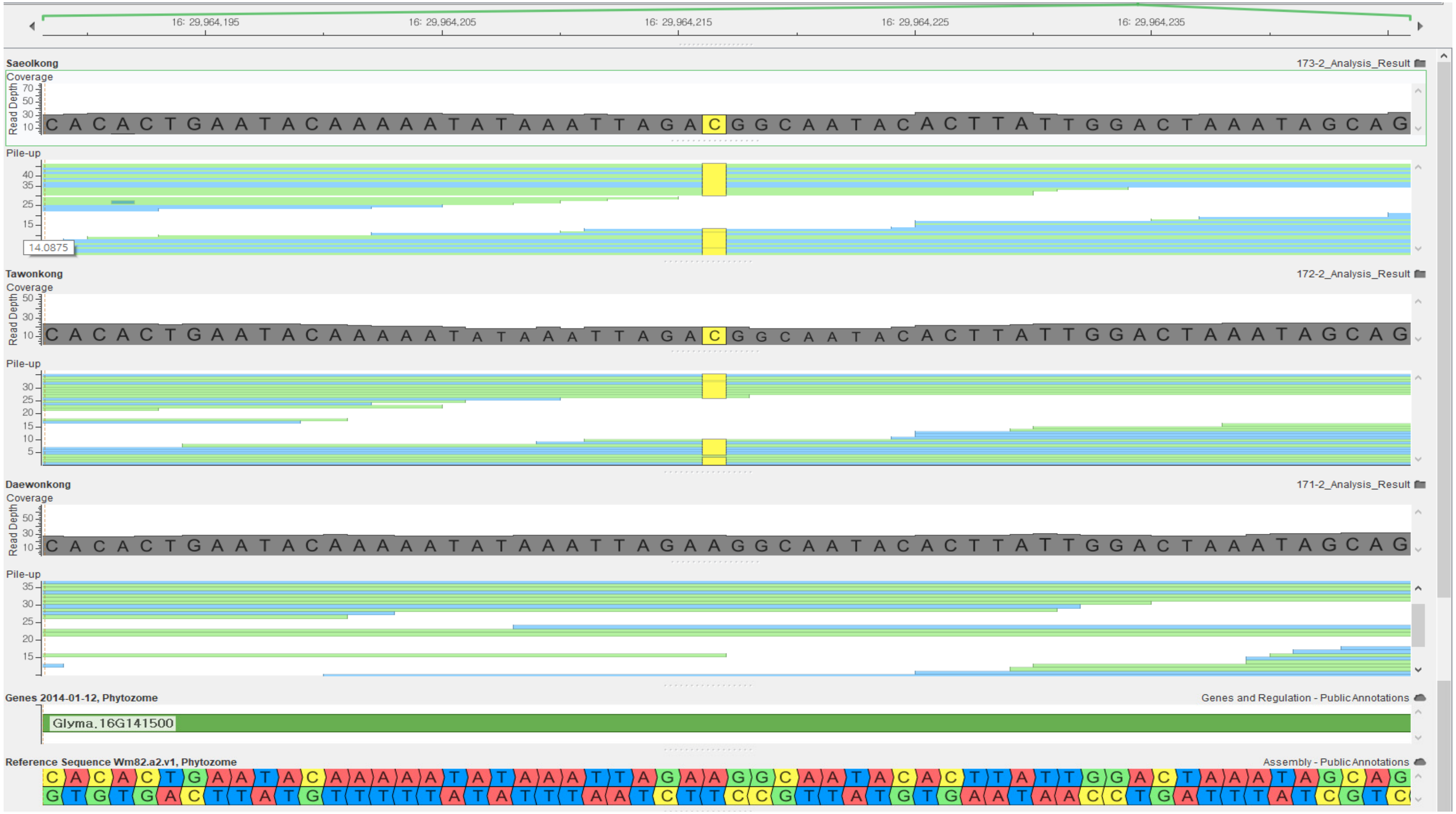

Figure S2. Target sequence of the *KASP-PS-2* marker (*Glyma.16g141500*, 1 bp mutation; A > C).

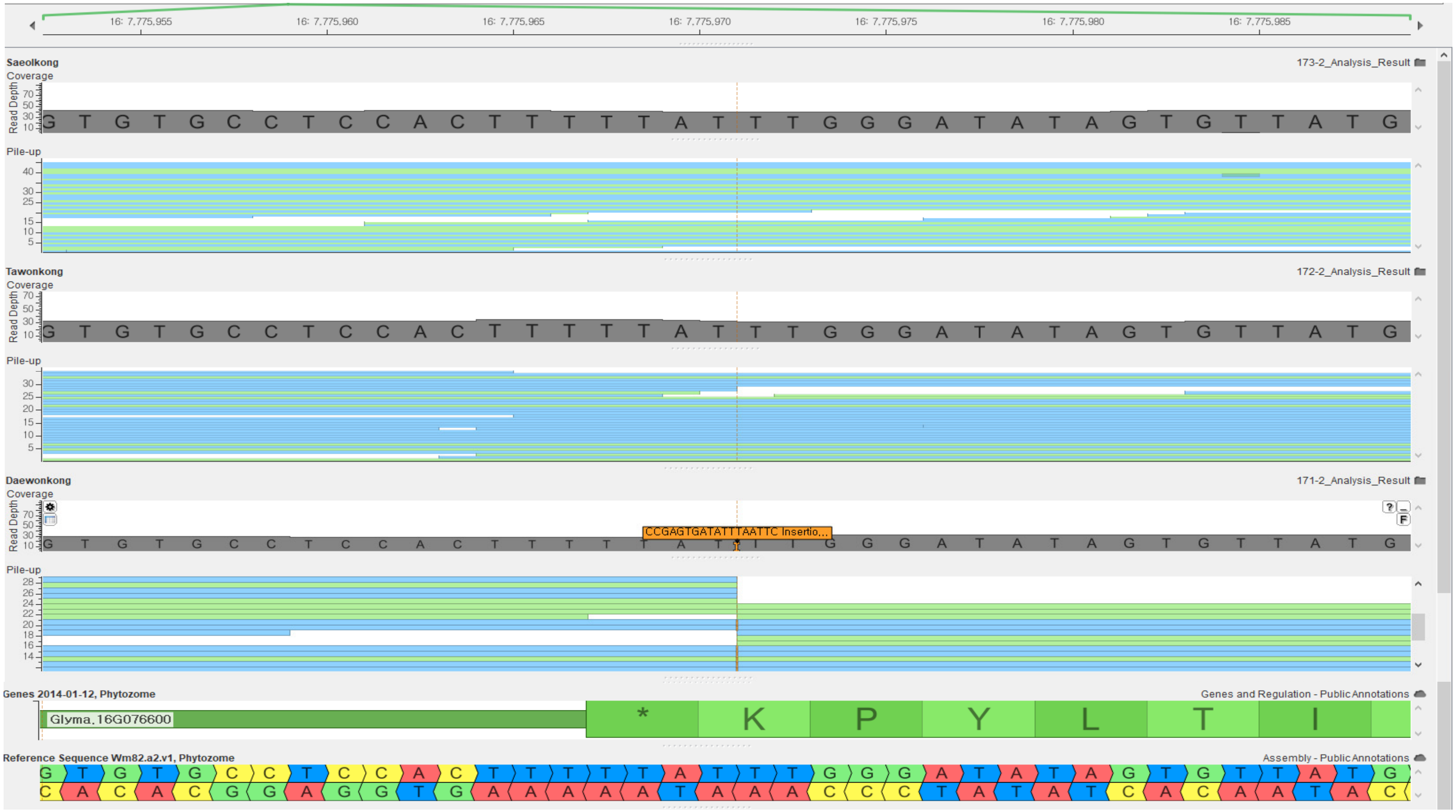

Figure S3. Target sequence of the InDel marker (*Glyma.16g076600*, 18 bp insertion).

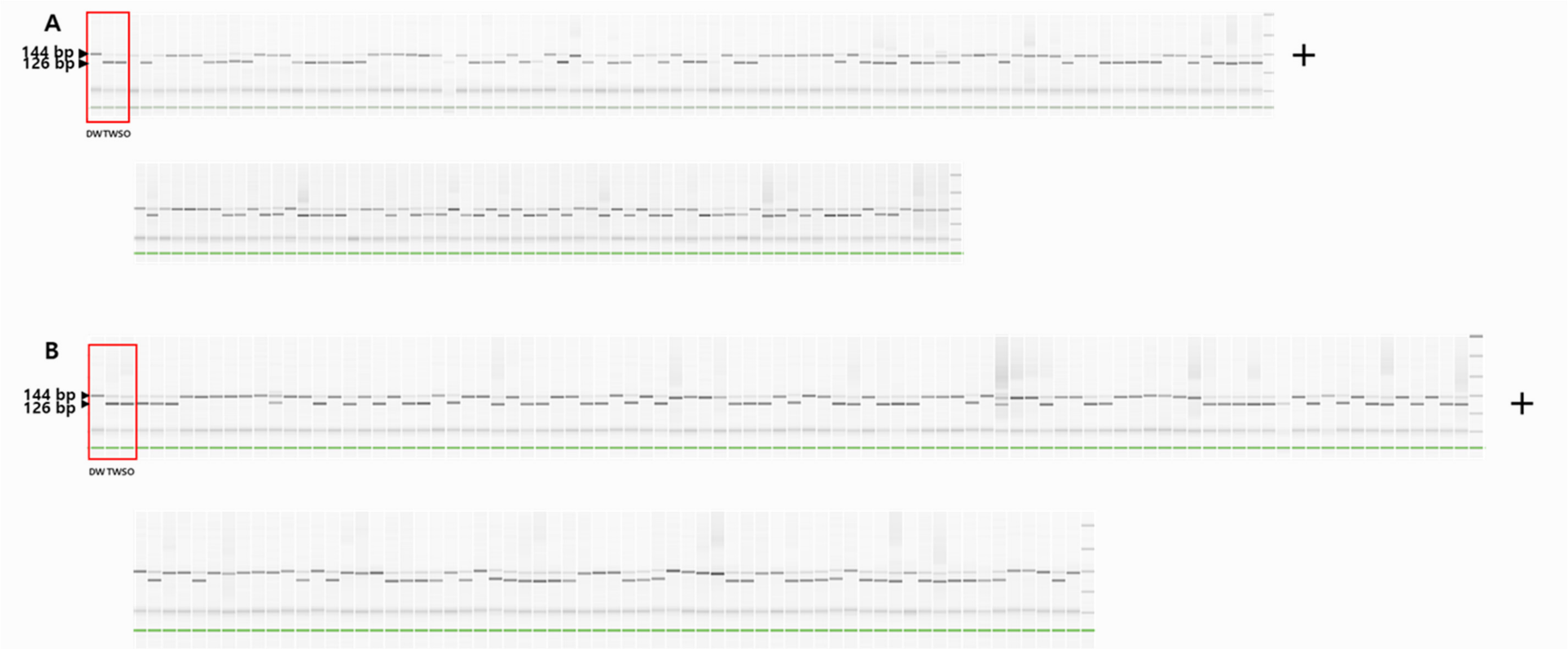

Fufure S4. Genotyping of the recombinant inbred line (RIL) populations using the InDel marker: (A) DT (Daewonkong × Tawonkong) population consisting 3 parental and 154 RILs and (B) DS (Daewonkong × Saeolkong) population consisting 3 parental and 153 RILs. DW, TW, and SO on the left indicates genotyping of Daewonkong, Tawonkong, and Saeolkong, respectively, and succeeding blocks are for RILs.

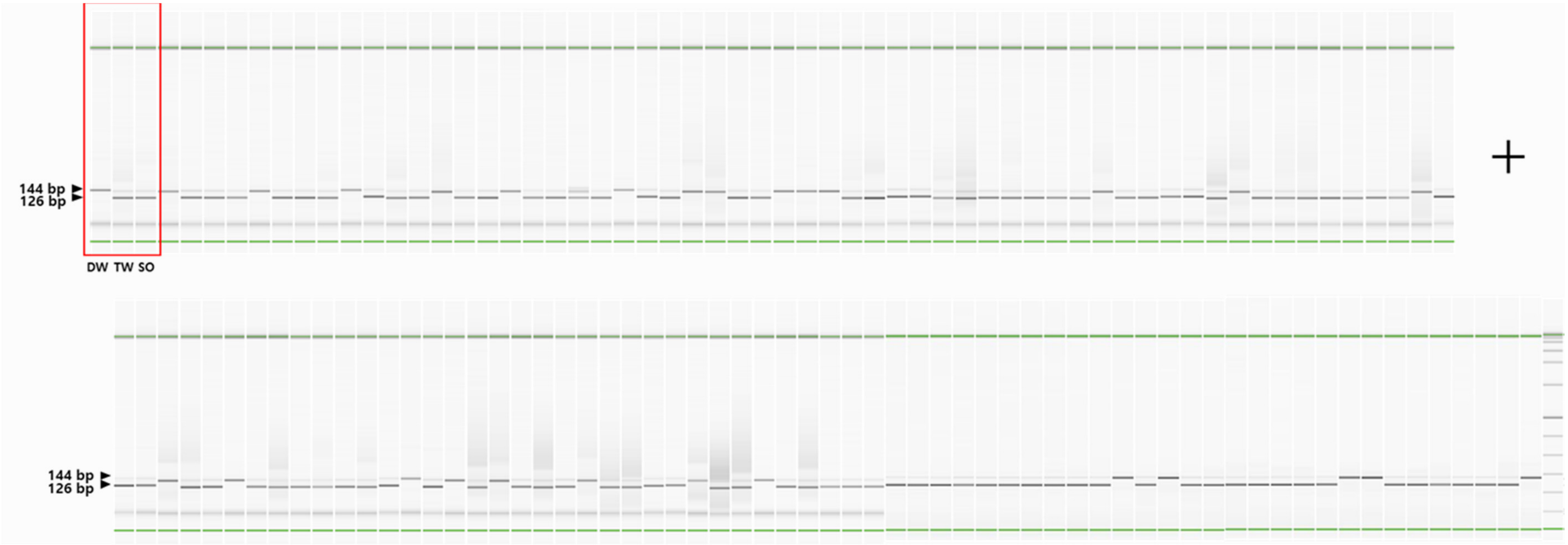

Figure S5. Genotyping of 120 varieties and elite lines using the InDel marker. DW, TW, and SO on the left indicates genotyping of Daewonkong, Tawonkong, and Saeolkong, respectively, and succeeding blocks are for the varieties and elite lines.

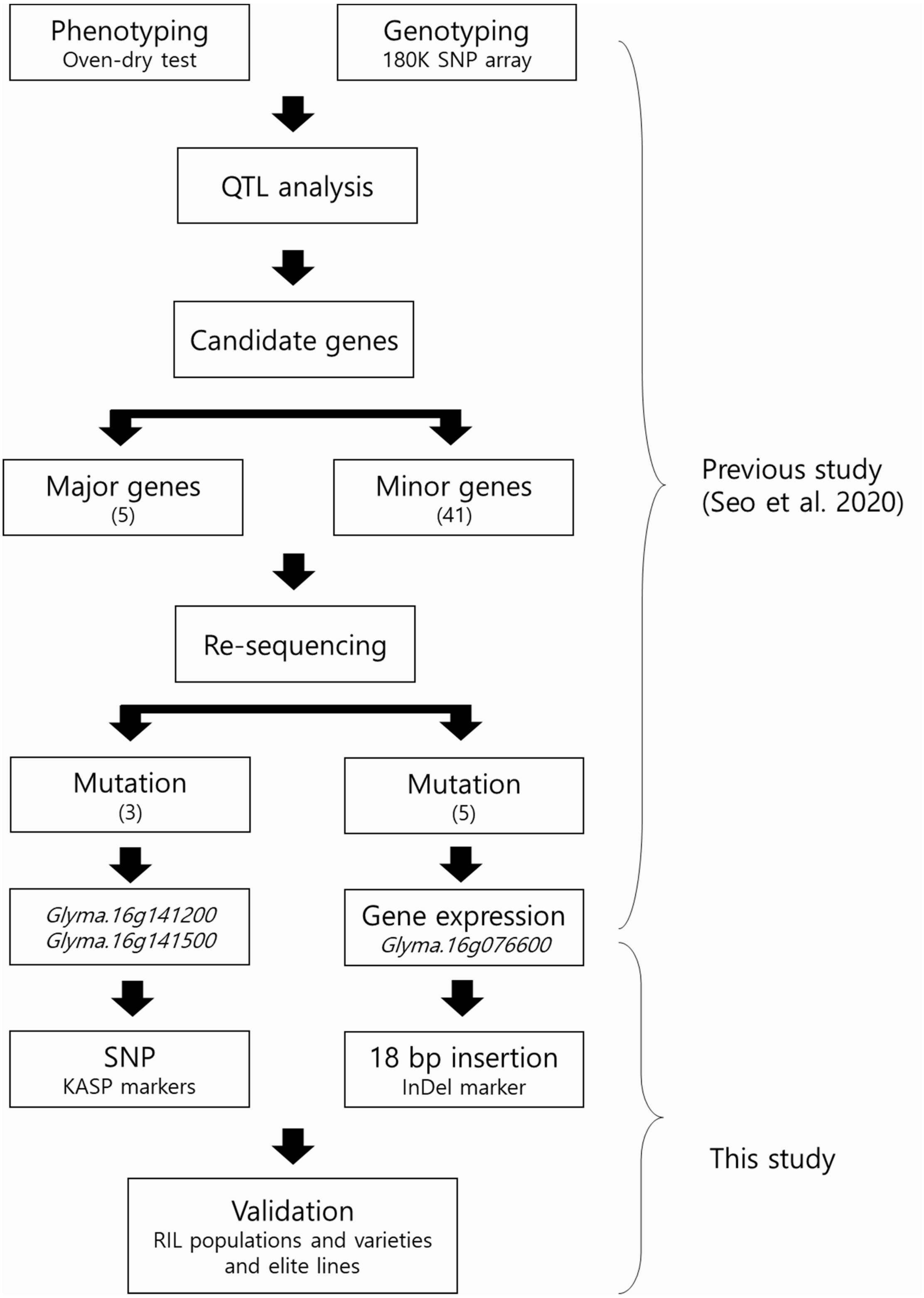

Figure S6. Schematic diagram of study design.

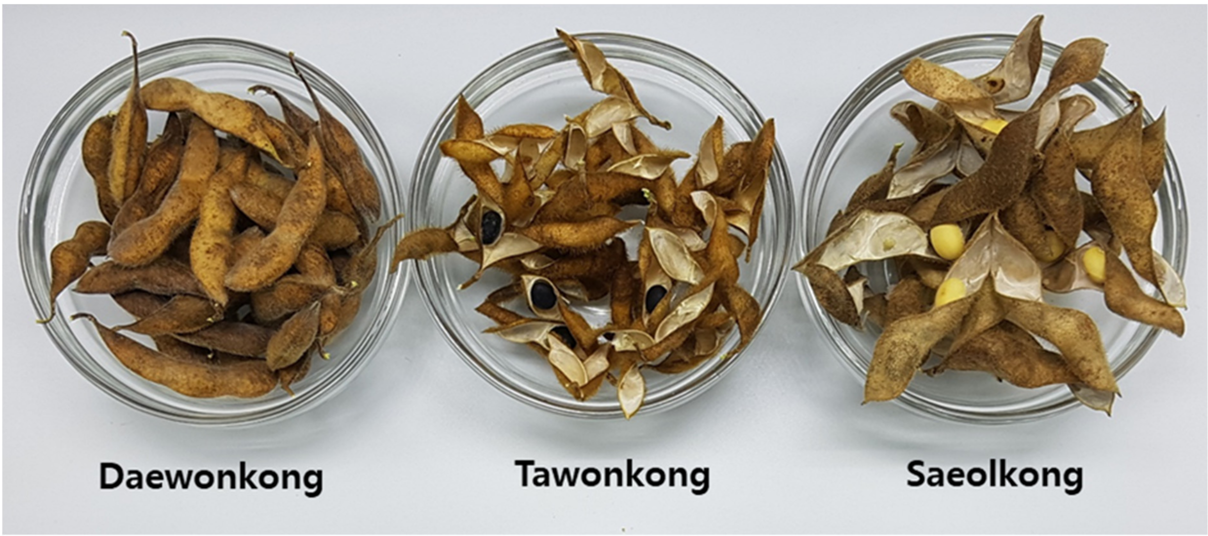

Figure S7. Pods of shattering-tolerant Daewonkong and shattering-sensitive Tanwonkong and Saeolkong after 72 hours of oven-drying.
